# Self-Expanding Metal Stents as an Alternative to Palliative Surgery in Advanced Obstructive Colorectal Cancer—A Systematic Review and Meta-Analysis

**DOI:** 10.3390/jcm14124339

**Published:** 2025-06-18

**Authors:** Vlad Rotaru, Elena Chitoran, Giuseppe Gullo, Daniela Viorica Mosoiu, Laurentiu Simion

**Affiliations:** 1 Medicine School, “Carol Davila” University of Medicine and Pharmacy, 050474 Bucharest, Romania; 2 General Surgery and Surgical Oncology Department I, Bucharest Institute of Oncology “Prof. Dr. Al. Trestioreanu”, 022328 Bucharest, Romania; 3 Department of Obstetrics and Gynecology, Villa Sofia Cervello Hospital, University of Palermo, 90146 Palermo, Italy; gullogiuseppe@libero.it; 4 Medicine School, “Transilvania” University, 500036 Brasov, Romania; 5 Hospice “Casa Sperantei”, 500074 Brasov, Romania

**Keywords:** self-expanding metal stents, obstructive colorectal cancer, palliative, stoma formation, intestinal obstruction

## Abstract

The diagnosis of colorectal cancer in more advanced stages, especially in younger patients where the diagnosis usually occurs because of obstructive complications, has prompted the development of less invasive, more rapid and well tolerated methods of decompression as an alternative to the standard surgical approach. As such, self-expanding metal stents (SEMSs) have gained wide acceptance for the palliative alleviation of obstructive symptoms in patients with advanced colorectal cancer. The purpose of this study was to evaluate SEMS placement against various forms of palliative surgical procedures in terms of effectiveness, morbidity, mortality and oncologic results. We conducted a systematic search of PubMed, Web of Science, Cochrane Library and Medline for articles describing patients with incurable locally advanced obstructive colorectal cancer who underwent surgery or self-expanding metal stent placement as a palliative procedure for the alleviation of symptoms. Eighteen studies (1606 patients) were included in a pooled meta-analysis. In the surgery group the clinical success was slightly higher (98.62% vs. 94.92%; OR = 0.35, 95%CI [0.16–0.73], *p* = 0.005) and the late complications rate was lower (13.9% vs. 24.0%; OR = 3.01, 95%CI [2.06–4.39], *p* < 0.00001). The SEMS placement was associated with a lower early complication (11.3% vs. 28.1%; OR = 0.34, 95%CI [0.19–0.58], *p* = 0.0001) and a shorter length of hospital stay (SMD = −1.94, 95%CI [−2.76, −1.12], *p* < 0.00001). In terms of the oncologic results, surgery was significantly associated with an increased overall survival regardless of the type of procedure (OR = 1.24, 95%CI [1.08–1.42], *p* = 0.002). Although having lower early morbidity and mortality rates, SEMS placement was associated with an increased chance of late complications and a worse overall survival, thus making them avoidable when patients have longer life expectancies. Due to the lower early complications rates, SEMSs might still have a place in the management of selected cases with bowel obstruction.

## 1. Introduction

Colorectal cancer (CRC) remains the third neoplastic disease in terms of incidence worldwide, with more than 1.9 million new cases each year [1]. In 2023, more than 60% of all colorectal tumors were diagnosed in advanced stages [2]. Also, because of modern living and external influences (e.g., dietary and lifestyle), there is a noticeable increasing tendency towards this illness manifesting itself in younger patients [3,4]. In such patients colorectal tumors are often diagnosed in more advanced stages, partially due to an overall lack of symptoms in the early stage of the disease and a general lower participation in screening methods than older patients. There is also a shift of the lateralization of tumors towards the left colon and rectum—left-sided colorectal cancer increased in incidence from 27% in 1995 to 31% in 2019 [2]. Left-sided colorectal cancers have a more pronounced tendency to cause obstructive phenomena [5].

Obstruction is a common complication of CRC and is often associated with advanced stages and with a severe prognosis [6,7], which will require an urgent decompressive intervention [8] to prevent increased intraabdominal pressure, electrolyte and acidotic disorders, septic complications and even death [9].

Advanced obstructive CRC (AO-CRC) is a particularly complex condition in which patients are often oriented to palliative or best supportive care, especially in the presence of associated severe medical conditions limiting curative possibilities or of irresectable tumors. For these patients, a surgical procedure (even a smaller palliative intervention) can further destabilize an already frail patient. An alternative to surgery that has gained wide acceptance and has become somewhat of a standard procedure for the alleviation of obstructive symptoms in patients with advanced CRC is the placement of self-expanding metal stents (SEMSs).

Although generally associated with better patient tolerability, immediate relief of obstructive symptoms, lower hospitalization lengths and minimal invasiveness, SEMSs also have specific complications that need to be considered when choosing such an approach. The most common stent-related complications are re-obstruction, stent migration or fracture, erosion, or even perforation of the digestive wall and the subsequent septic complications and intraluminal cancer cell dissemination. Moreover, long-term complications, such as bleeding and functional disorders, like pain, diarrhea and tenesmus, limit SEMS utilization in patients with long life expectancies. In patients with AO-CRC that are eligible for adjuvant therapies and with long life expectancies, other palliative options of decompression should be considered. Some surgical procedures, like resection of the primary tumor, have been suggested to lead to a better overall survival and improved quality of life [10,11,12].

Despite the considerations above, there is very little literature available describing the direct outcome comparisons between various palliative procedures for AO-CRC. Previous meta-analyses tried to evaluate the comparative results of surgery and a SEMS for malignant obstruction, but the vast majority also included obstructions of the digestive tract caused by urological/gynecologic cancers or where a SEMS was used as a bridge procedure prior to neoadjuvant therapy and radical surgery [13,14,15]. Some previous meta-analyses described the survival results in terms of the shear duration [13,16], whereas we considered that odds ratios (ORs) are better suited to compare treatment methods.

To address the aforementioned knowledge gaps, we conducted an extensive literature review and performed a meta-analysis to examine SEMS placement against various forms of palliative surgical procedures. Moreover, we conducted a subgroup analysis, which allowed us to draw conclusions on how SEMSs compare with specific surgical procedures in palliative settings.

## 2. Materials and Methods

### 2.1. Search Strategy

We conducted a search of four international databases (PubMed, Web of Science, Cochrane Library and Medline) for articles describing adult patients with incurable locally advanced obstructive colorectal cancer who underwent surgery or self-expanding metal stent placement as a palliative procedure for the alleviation of symptoms and providing conditions for the rapid initiation of systemic therapy. The search was conducted using a combination of Boolean coordinators and keywords, including “colorectal cancer”, “locally advanced”, “obstructive/obstruction”, “palliative”, “surgery”, “stent” and “stoma”. All studies were considered regardless of the year of publication (from the inception of the databases and up to January 2025). Additional relevant studies were identified through checking the reference lists of included studies to identify any other relevant articles.

The exclusion criteria included the following: studies in which self-expanding metal stents/surgical procedures were intended as a bridge procedure before neoadjuvant therapy and radical surgery; studies that included patients without intestinal obstruction or with obstructions caused by other causes than colorectal tumors; single-arm studies or studies in which no comparison of self-expanding metal stents with another procedure were available; and systematic reviews and meta-analyses.

The outcomes of interest included the following: clinical success of the procedure (defined as the alleviation of obstructive symptoms), early complications (defined as complications that occurred within 30 days from the palliative intervention), late complications (defined as a procedure related complications that occurred after 30 days), mortality, overall survival rate and length of hospital stay. Table 1 presents the PICOS criteria (Population, Intervention, Comparison, Outcomes and Study framework for systematic reviews) used in our search strategy.

### 2.2. Data Extraction

All resulting articles were processed; duplicates and articles presenting previously published results were removed. Two authors (E.C. and V.R.) performed an initial screening for the suitability of the titles and abstracts of the studies. The studies considered eligible by both authors were included. The differences were then settled by consulting with the third author (L.S.). The remaining articles were then sought for full-text retrieval. A second screening was then performed, and ineligible articles were excluded. The reasons for exclusions were non-obstructive tumors included, obstruction caused by causes other than colorectal cancer, the type of article (reviews or meta-analysis), no outcomes of interest or no comparison of outcomes available.

### 2.3. Bias Assessment of Included Studies

The included studies were evaluated for quality and risk of bias using the Cochrane risk of bias tool for randomized control trials and the Newcastle–Ottawa scale for the non-randomized studies. The results are summarized in Table 2.

### 2.4. Statistical Analysis

All analyses were conducted using the open-source Review Manager 5.4 software. The chi-square test and Student’s *t*-test were used to compare the differences between the two sub-groups, and a *p*-value lower than 0.05 was considered statistically significant. For categorical variables, the odds ratios (ORs) and their respective 95% confidence intervals (95%CI) were used for the analysis and comparison. Continuous variables were analyzed using the standardized mean difference (SMD) and confidence intervals. The 95% confidence intervals for the OR that did not cross 1 and for the SMD that did not cross 0 were considered statistically significant. The pooled results were then presented as forest plots. A sensitivity analysis was also performed to evaluate the heterogeneity. The heterogeneity of the studies was assessed using the *I*^2^. An *I*^2^ > 50% was considered indicative of heterogeneity. When heterogeneity was proven, the risk of bias was mitigated by using a random-effects model for the analysis. Fixed-effects models were used for low heterogeneity. For results that exhibited heterogeneity, subgroup analysis was performed by publication year, tumor location and type of study. Further subgroup analysis was performed by the type of intervention self-expanding metal stents (SEMSs) vs. resection of primary tumor (RPT) and vs. stoma formation/internal bypass (SF/IB) in order to discern whether any differences occurred when using a SEMS compared with standard surgical procedures.

## 3. Results

**Figure 1:**
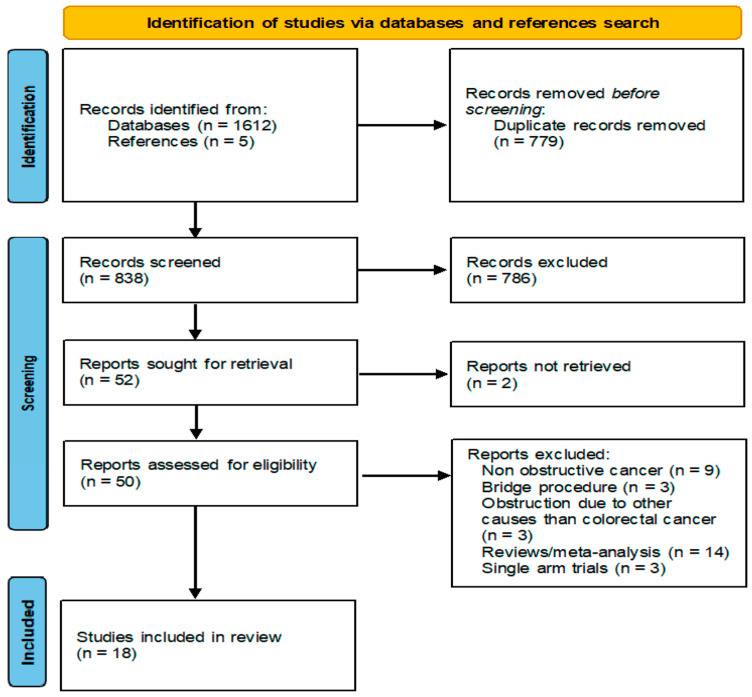
Search flow diagram.

Through the database search, a total of 1612 records were identified. An additional five studies were identified via a manual search for relevant references. A total of 779 duplicates were removed via automatic methods (Mendeley Reference Manager). The remaining 838 studies were screened by title and abstract for eligibility and 786 were excluded due to an irrelevant focus. We were able to procure the full texts for 50 out of 52 studies sought for retrieval. After the full-text screening, 32 studies were excluded for various reasons (9—the colorectal cancer was not obstructive, 3—described stenoses due to other tumors than colorectal cancer, 3—the intervention was used as a bridge procedure before neoadjuvant therapy and radical surgery, 14—the studies were review-type or a meta-analysis and 3—the studies described single-arm studies). The final 18 studies published between 2003 and 2022, which encompassed 1606 patients (791 had a SEMS placed as a palliative procedure and 815 underwent an alternative surgical procedure, such as stoma formation, internal bypass or palliative primary tumor resection), were included in this review. Six studies compared the SEMS placement with a specific surgical procedure (3—SEMS vs. SF/IB, 3—SEMS vs. RPT). In total, 2 of the included studies were randomized controlled trials and 16 were non-randomized (5 prospective in nature and 11 retrospective). The screening process is presented in Figure 1 and the main characteristics of the included studies (including the evaluation of the bias risk using the Newcastle–Ottawa scale or the Cochrane tools) are detailed in Table 2.

Seventeen studies provided information on complications associated with SEMS placement by describing stent-related complications (such as perforation, migration, narrowing of the channel and rupture of the stent), re-obstruction, functional disorders (such as pain or diarrhea), hemorrhagic complication (actual bleeding or anemia), infectious complications (wound infections, abscesses, urinary or pulmonary infections, or sepsis/bacteremia) and thromboembolic events, and culminating with organ failure. Similarly, 15 studies described the complications associated with surgical procedures (stoma-related, surgery-related and all general complications described for SEMSs).

### 3.1. Outcome Analysis

**Figure 2:**
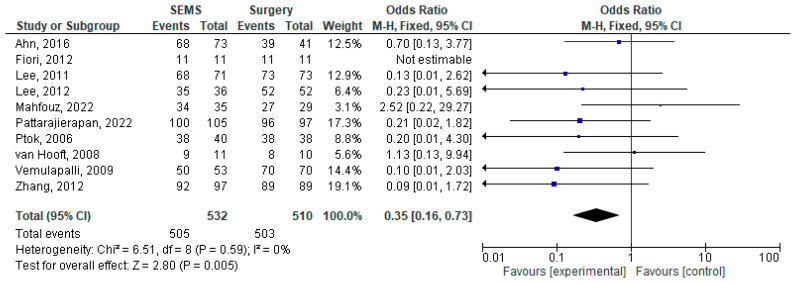
Forest plot of outcome: clinical success [17,22,23,26,27,28,29,30,33,34].

**Figure 3:**
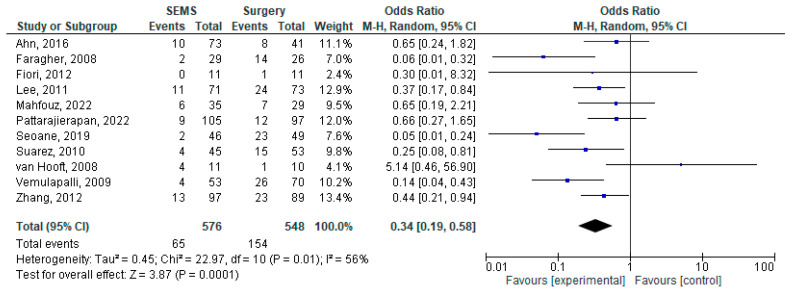
Forest plot of outcome: early complications (<30 days from intervention) [17,20,22,23,26,28,29,31,32,33,34].

**Figure 4:**
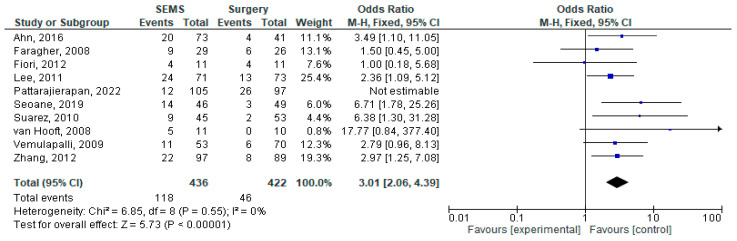
Forest plot of outcome: late complication [17,20,22,23,26,29,31,32,33,34].

**Figure 5:**
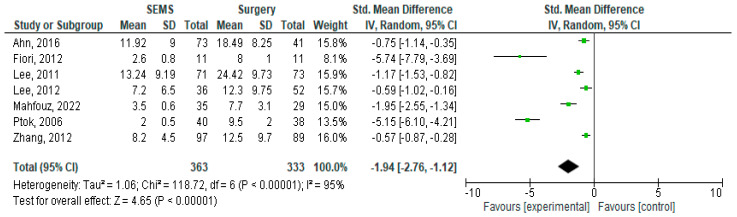
Forest plot of outcome: length of hospital stay [17,22,26,27,28,30,34].

**Figure 6:**
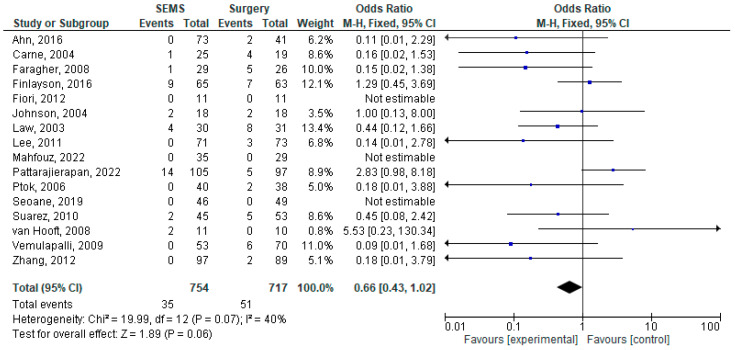
Forest plot of outcome: early mortality (<30 days from intervention) [17,18,20,21,22,23,24,25,26,28,29,30,31,32,33,34].

**Figure 7:**
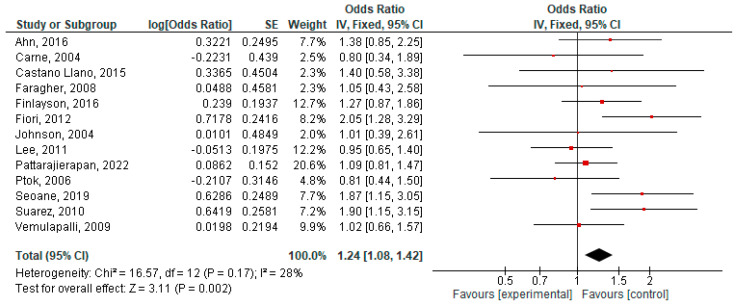
Forest plot of outcome: overall survival [17,18,19,20,21,22,24,26,29,30,31,32,33].

The results of the meta-analysis are presented as forest plots in Figure 2, Figure 3, Figure 4, Figure 5, Figure 6 and Figure 7.

The clinical success of the intervention was reported by 10 studies [17,22,23,26,27,28,29,30,33,34] (1042 patients) and the pooled results showed an overall success rate of 96.74% (1008 patients). In terms of frequencies, the clinical success was slightly higher in the surgery group (98.62% vs. 94.92%). The meta-analysis of this outcome used a fixed-effects model and showed a corresponding result (OR = 0.35, 95%CI [0.16–0.73], *p* = 0.005). On the other hand, the stoma formation rate was higher in the surgical patients (52.99% vs. 11.68%; OR = 0.11, 95%CI [0.05–0.22], *p* < 0.01).

The pooled analysis of the early and late complications was performed using random-effects models due to high heterogeneity between the 11 studies reporting them [17,20,22,23,26,28,29,31,32,33,34]. Early complications were less frequent in the SEMS group when compared with the surgical approaches (among the 576 patients in the SEMS group, 65 experienced such complications—11.3% vs. 154 patients out of 548 in surgery group—28.1%). The difference was statistically significant and was confirmed by the meta-analysis, which showed an OR = 0.34 (95%CI [0.19–0.58], *p* = 0.0001). This result may be explained by the added stress of general anesthesia and the subsequent immunosuppression. However, when analyzing the late complications, the situation was reversed with a higher incidence in the SEMS group (24.0% vs. 13.9% in the surgical group). The OR calculated in the meta-analysis was statistically significant at a value of 2.3 (95%CI [1.22–4.36], *p* < 0.01).

Almost all studies reported the 30-day mortality rate (16 studies [17,18,20,21,22,23,24,25,26,28,29,30,31,32,33,34] that enrolled 1473 patients). The SEMS group seemed to have a lower early mortality frequency than the surgical group (4.64% vs. 7.11%) but the result was not significant. A pooled analysis showed OR = 0.66, 95%CI [0.43–1.02], *p* = 0.06.

Seven studies offered information on the length of hospitalization of the two groups [15,16,17,23,29,33,34]. As expected, pooled analysis showed a longer hospitalization for the surgical group. The calculated standard mean difference was −1.94, 95%CI [−2.76, −1.12], *p* < 0.00001. We must mention the extreme heterogeneity of these seven studies with an *I^2^* value of 95%, which made us use a random-effects model for the analysis.

Finally, we wanted to evaluate which type of palliative procedure used for decompression influenced the overall survival of the patients in any way. The pooled results from 13 different studies [17,18,19,20,21,22,24,26,29,30,31,32,33] show an odds ratio (OR) of 1.24, 95%CI [1.08–1.42], favoring the surgical group, and this result was significant (*p* = 0.002).

### 3.2. Heterogeneity, Publication Bias and Sensitivity Analysis

**Figure 8:**
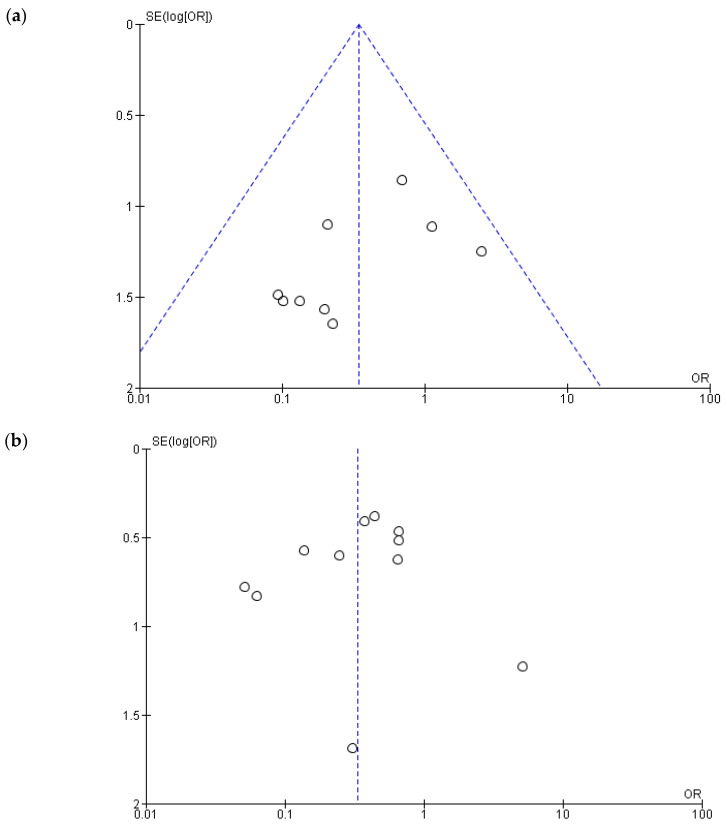

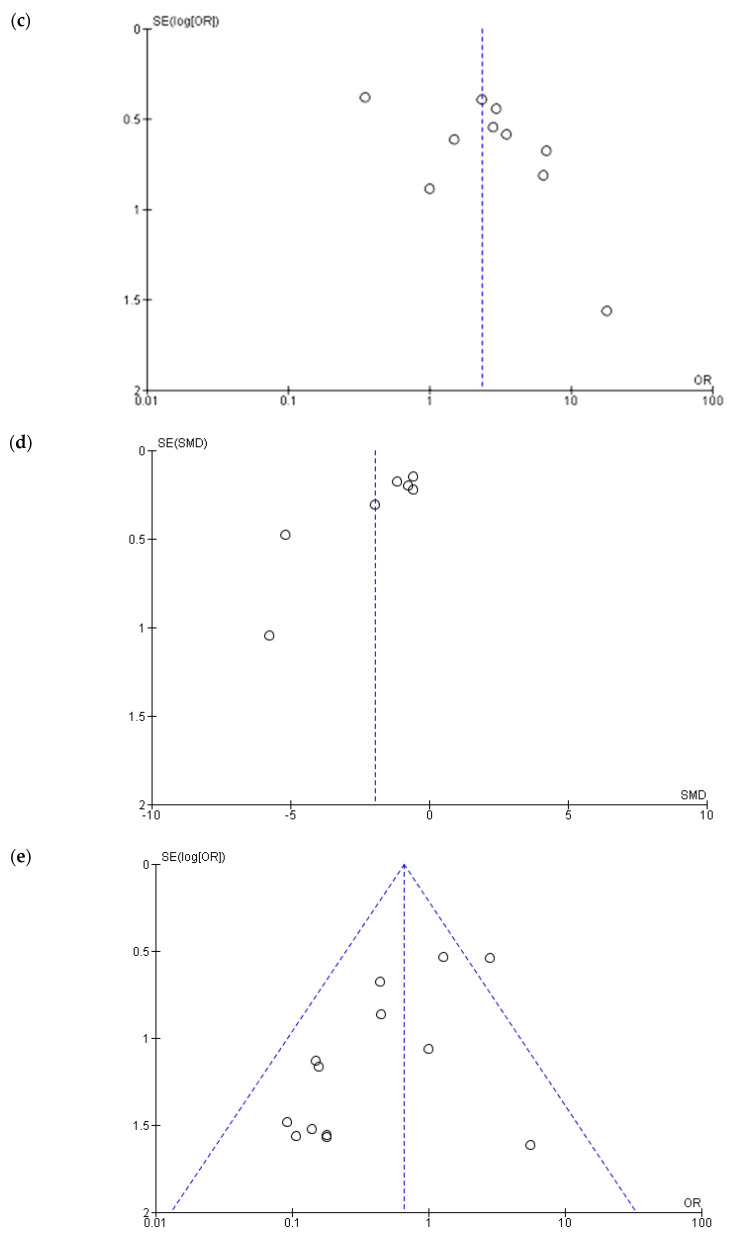

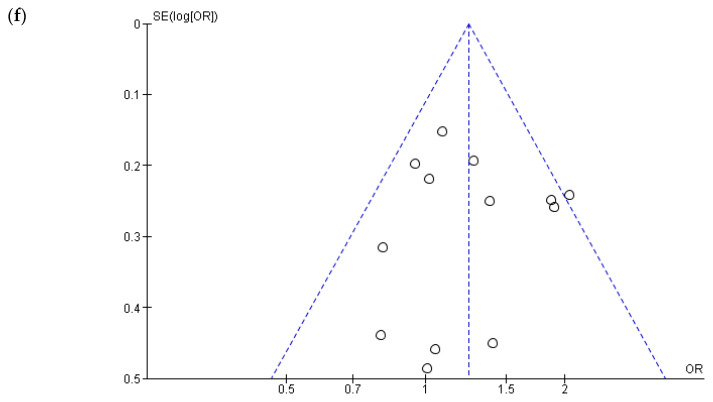
Heterogeneity and publication bias—funnel plots: (**a**) clinical success; (**b**) early complications (within 30 days from intervention); (**c**) late complication; (**d**) length of hospital stay; (**e**) early mortality (within 30 days from intervention); (**f**) overall survival.

For three of the six studied outcomes, we found evidence of heterogeneity across the included studies (the funnel plots for all the studied outcomes are presented in Figure 8): early complications—*I*^2^ = 56%, late complications—*I*^2^ = 71% and length of hospital stay—*I*^2^ = 95%. The calculated *P*_egger_ values for the early and late complications were >0.1, indicating that no significant publication bias existed and heterogeneity may have been caused by other factors. However, for the length of hospital stay, the value of *P*_egger_ was 0.01, thus indicating that the heterogeneity may have been due to publication bias.

For the three results that exhibited high heterogeneity, we performed a sensitivity analysis by excluding each individual study to evaluate whether any single study had a significant impact on the pooled results. We found no major impact of any of the studies on the pooled results for early complications and length of hospital stay. However, when removing the Pattarajierapan (2022) [29] study from the analysis for late complications, we found that the heterogeneity was reduced to 0%—thus indicating a high impact of this study on the overall result for this outcome. The Pattarajierapan study [29] compared a SEMS and SF/IB and our subgroup analysis suggested that SF/IB may have been associated with a higher rate of late complications than other surgical procedures, which could explain at least in part the source of heterogeneity. Additionally, this study focused on Asian patients, thus racial differences may have also contributed to the heterogeneity.

### 3.3. Subgroup Analysis

For the results that show high heterogeneity, we performed a subgroup analysis to provide additional insights based on the following criteria: publication year ≤2010 or >2010, type of study (prospective vs. retrospective vs. randomized controlled trials), left colorectal cancer vs. all types of colorectal cancer and type of surgical procedure (SF/IB vs. RPT vs. not specified surgical procedure). We were unable to perform an additional subgroup analysis based on the age of the patients, functional status or prior systemic therapy due to the unavailability of usable data in the included studies. The results are presented in Table 3, where we highlighted results that are statistically significant while simultaneously not exhibiting high heterogeneity (*I*^2^ < 50%).

### 3.4. Early Complications

During our subgroup analysis of complications that occurred during the first 30 days after the palliative procedure, we found that in the studies published after 2010, there was a higher probability of lower early complications rate for the SEMS group compared with the surgical group (*I*^2^ = 39%, OR = 0.42, 95%CI [0.25, 0.71], *p*-value < 0.001). The studies of retrospective nature were also associated with lower early complications rates in the SEMS group (*I*^2^ = 41%, OR = 0.33, 95%CI [0.19, 0.55], *p*-value < 0.0001). There was evidence of higher early complications rates in surgical groups when we compared the patients that had a SEMS placement with patients that underwent resection of a primary tumor (RPT). Stoma formation or surgical internal bypasses were not associated with a significant difference in the early complication rates when compared with the SEMS placement.

### 3.5. Late Complications

Late complication rates were found to be significantly influenced by the year of publication, type of study and type of surgical procedure. The surgical group was less likely to develop late complications than the SEMS placement group in the studies published up to and including 2010 (*I*^2^ = 14%, OR = 3.03, 95%CI [1.39, 6.56], *p*-value <0.005), in retrospective studies (*I*^2^ = 0%, OR = 2.73, 95%CI [1.79, 4.15], *p*-value < 0.00001) or when SEMS placement was compared with SF/IB or a heterogenous group of surgical procedures including SF/IB (respectively, *I*^2^ = 14%, OR = 0.44, 95%CI [0.19, 1.00], *p*-value < 0.05; *I*^2^ = 0%, OR = 3.34, 95%CI [2.11, 5.29], *p*-value < 0.00001).

### 3.6. Length of Hospital Stay

The subgroup analysis could not identify any relevant factors that could explain the heterogeneity between the included studies.

## 4. Discussion

The past decade’s shift of colorectal cancer towards diagnosis in more advanced stages, especially in younger patients, where diagnosis usually occurs as a result of obstructive complications, has prompted the development of less invasive, more rapid and well tolerated methods of decompression as an alternative to the standard surgical approach. As such, SEMS gained wide acceptance and became somewhat of a standard for palliative alleviation of obstructive symptoms in patients with advanced colorectal cancer.

The success rate for the alleviation of obstructive symptoms of a SEMS was found to be 94.92%, which was slightly lower than in the surgical group (98.62%), yet consistent with previous published studies [35,36]. The main reasons involved in SEMS failure were stent-related complications [37], such as migration and re-obstruction, or the presence of peritoneal metastatic disease [35,38]. Other risk factors described as influencing the clinical success rate of a SEMS was colorectal compression due to extrinsic causes (such as gynecologic and urologic cancers) and the actual length of the stent used [39]. Surgery offers additional possibilities when dealing with carcinomatosis, thus improving the chances of successful decompression. The success rate of SEMS placement may be improved by direct endoscopic visualization of the stenotic area, which resulted in an improved success rate (81% vs. 77%) and fewer complications (20% vs. 38%) than when the SEMS was placed under radiographic guidance [40]. On the other hand, the stoma formation rate was higher in the surgical patients (52.99% vs. 11.68), but this result was easily explainable by the actual surgical possibilities versus endoscopic procedure.

Another factor to consider when choosing a decompression method for patients with AO-CRC is biologic conditions. Frail patients or patients with heavy medical comorbidities need to be protected from the impact of general anesthesia and post-operative immunosuppression. In other cases, the extent of the neoplastic disease may require salvage systemic therapy to improve the odds, which would only be delayed by the post-operative recovery. With these considerations in mind, SEMS placement becomes a logical alternative to surgery for these patients [17,21,26,29,31]. Some authors have raised some concerns about using SEMSs in patients that will require chemotherapy plus targeted therapies, such as Bevacizumab, due to the increased risk of ischemic perforation [41]. Although a SEMS in association with chemotherapy plus Bevacizumab increases the risk for perforations (63.4% vs. 25.7%), this combined approach increased survival, as proven by a review that enrolled more than 680 patients [42]. In patients with AO-CRC that will undergo combined chemotherapy and targeted immunotherapy, a SEMS should be considered carefully, weighing the potential benefits of the fast alleviation of obstructive symptoms and quick initiation of therapy to potential stent-related complications (some of them favored by the targeted therapies). However, SEMS should not be completely ruled out due to survival benefits that are suggested by some studies.

SEMS-specific complications (the most common being re-obstruction, perforation and migration) need to be considered when employed as a palliative procedure for AO-CRC since these complications may lead to poor overall prognostic and reduced post-operative survival [40,43]. Early re-obstruction usually occurs due to failure to complete the expansion, whereas late re-obstruction is caused by tumor growth inside the stent’s lumen. A stent expansion of less than 70% within 2 days from placement is statistically significantly associated with early stent occlusion (OR = 12.5, 95%CI [2.25–62.48]) [44]. Late tumor ingrowth may be treated by endoscopic electrocoagulation and the replacement of the stent/placement of a secondary stent inside the existing one, but usually the success rate is around 75–85% [45]. These are effective treatment options when re-obstruction occurs, which may provide patency until the end of life in palliative settings. The median patency of SEMSs has been reported to be between 55 and 340 days in palliative settings [45,46], and this period needs to be considered in balance with the expected length of survival. Our study also confirmed that a SEMS may not be an optimal choice for patients in which we expect longer survival—the results suggest that SEMS was associated with a reduced overall survival (OR = 1.24 favoring the surgical group, *p* = 0.002). These results contradict the results of some previous meta-analyses [13,15,16], but are consistent with other studies [17,21,31,32]. The re-obstruction rate may be reduced by using a covered stent, but such stents may increase the risk of migration [47,48]. The most severe complication of SEMS placement is perforation and secondary peritonitis, requiring emergency surgery and often results in stoma formation [46]. In patients with AO-CRC, the bowel preparation is usually poor, and the quality of the intestinal wall is weak, especially around the tumoral narrowing. These factors contribute to an increased risk of perforation via erosion of the tumoral colorectal wall or ischemic lesions. Unlike biodegradable or polymeric stents, metal stents (titanium or stainless steel) may cause foreign body reactions, which can cause secondary perforation; however, in terms of strong support, they are superior.

The present meta-analysis suggested that SEMS placement as a palliative procedure in AO-CRC is associated with fewer early complications than surgical procedures (11.3% vs. 28.1%) and a shorter length of hospital stays. This result may be explained by the added stress of general anesthesia and the subsequent immunosuppression. However, when analyzing late complications, the situation was reversed with a higher incidence in the SEMS group (24.0% vs. 13.9% in the surgical group), which was explainable by stent migration, perforation or re-obstruction. Although SEMSs seem to have lower early mortality rates than the surgical group (4.64% vs. 7.11%), the association was not significant. These results, consistent with those of previous studies, highlight the need for additional comprehensive studies that focus on the impact of SEMS placement compared with a classical surgical approach in both immediate and extended postprocedural timelines.

The clinical success rate (alleviation of obstructive symptoms) was slightly higher in the surgery group (98.62% vs. 94.92%). On the other hand, the stoma formation rate was higher in the surgical patients (52.99% vs. 11.68%), but this result was easily explainable by the actual surgical possibilities versus endoscopic procedure (for very low rectal tumors, a successful surgical anastomosis is highly improbable, and stoma formation becomes unavoidable). For patients with AO-CRC, which are usually frail, the treatment option should be chosen after taking into account factors such as the patient medical condition, histology, systemic and local tumoral extensions, hospital facilities and personal preferences of the patients. The present study suggests better clinical success rates, improved survival and fewer late complications. And although surgical results are only partially superior to SEMS placement, our study provides a basis for individualized treatment and a focus on improved quality of life, like many more other procedures in surgical oncology [49,50,51,52].

Resection of the primary tumor seems to have better survival results when compared with a SEMS but also with other surgical procedures, such as stoma formation or internal bypasses, but it requires careful patient selection and making sure the patient can in fact support the surgical procedure. These may have been due to the fact that the resection of the primary tumor effectively erased the uncertainty introduced by the continuous tumoral growth (both local and systematic dissemination), which the SEMS did not, although it was able to rapidly alleviate obstructive symptoms. We must mention that in the studies included in our meta-analysis, the surgical procedure was mostly selected by the attending surgeon according to their own experience/preference and intraoperative conditions [17,18,19,20,21,23,25,30,32,33,34]. This type of selection of surgical procedures introduces important unpredictability into patient prognosis and is a serious limitation of the existing literature. According to our subgroup analysis, the resection of a primary tumor is the only type of open procedure associated with higher early complication rates than SEMS placement. Bypasses and surgical colostomies do not seem to be associated with higher complication rates. This aspect may be explained by the fact that the resection of the primary tumor usually involves more extensive dissections and longer periods of anesthesia. Our results further highlight the importance of the selection of patients in all aspects of oncologic treatment, which is in full concordance with other studies on various tumors and various therapeutical options [46,47,48,49,50].

### Study Limitations

The greatest limitation of our study was that thus far, there are not sufficient good quality studies on the impact of SEMSs as palliative procedures in AO-CRC. Here were a limited number of relevant studies, which led us to including older studies, and this was another limitation of our analysis with possible implications for the results (as shown in the subgroup analysis). Randomized controlled trials are extremely rare. There is a pronounced need for additional high-quality randomized controlled trials that study SEMS efficacity and safety but also consider the differences in baseline patient status and tumor conditions across groups. Long-term follow-up is necessary to provide additional insights into overall survival of patients with AO-CRC. High-quality research is essential for validating our research and for establishing a common evidence-based guideline for selecting the best method for decompression in AO-CRC. It is also important that future research clarifies the suggested superiority of surgical methods like resection of primary tumors in terms of overall survival.

The heterogeneity between the existing studies is high, and although we tried to explain it through a rigorous subgroup analysis, some heterogeneity remains unexplained.

Another limitation of our study was that the methodology for this systematic review and meta-analysis was not registered prior to data gathering.

## 5. Conclusions

Although having lower early morbidity and mortality rates, SEMS placement is associated with an increased chance of late complications and a worse overall survival, thus making them avoidable when patients have longer life expectancies. Yet, with a similar clinical success rate than usual surgical palliative procedures, a SEMS can provide an optimal alternative for the rapid alleviation of obstructive symptoms, especially in medically complex cases or patients requiring salvage systemic therapy. SEMS placement may be inferior in terms of overall survival as a palliative decompressive procedure to resect a primary tumor. Our results indicate that in patients with AO-CRC, neither surgery nor SEMS should be ruled out and a personalized treatment plan should be designed for each patient considering the biological condition of the patient; comorbidities; need for chemotherapy and targeted agents; risk factors (such as the presence of carcinomatosis); and not lastly, the patient’s preference and physician’s experience.

## Figures and Tables

**Table 1:** PICOS criteria for inclusion of trials.

Parameter	Inclusion Criteria
Participants	Patients with incurable locally advanced obstructive colorectal cancer
Intervention	Self-expanding metal stents (SEMSs) placement as a palliative procedure
Comparison	Various alternative surgical approaches to SEMSs
Outcomes	Clinical success of the procedure Early and late procedure-related complications Early mortality Overall survival rate Length of hospital stay
Study design	Randomized or non-randomized controlled trials

**Table 2:** Characteristics of included studies.

Study	Type of Study, Study Population	Observation	Sample Size and Type of Palliative Procedure	Reported Complications		Risk of Bias (Newcastle–Ottawa Scale/Cochrane Tool)
				SEMS	Surgery	
Ahn, H.J. (South Korea, 2016) [17]	Retrospective, patients with unresectable CRC obstruction, ECOG 0–3.	Surgery may be preferable to SEMS in ECOG 0–1 patients. No differences in survival were observed in ECOG 2–3 patients.	N = 114 SEMS (n = 73) Surgery (n = 41)	Stent related Obstruction/ileus	Obstruction/ileus Surgery related Infectious complications	7
Carne, P.W.G. (New Zealand, 2004) [18]	Retrospective, patients with unresectable CRC obstruction, ASA 1–4, SEMS group: median age, 66 (range, 37–88) years, 13 males. Surgery group: median age, 68 (range, 51–85) years, 12 males.	Patients treated with stents were discharged earlier than after surgery Stents did not affect survival.	N = 44 SEMS (n = 25) Surgery (n = 19)	Stent related Obstruction/ileus Functional disorders	-	5
Castano Llano, R. (Colombia, 2015) [19]	Retrospective, patients with unresectable CRC obstruction in all stages of disease; 49,5% males vs. 50,5% females; median age 60 years (range, 28–87).	Stenting appeared to reduce the need for stoma formation.	N = 47 SEMS (n = 24) Surgery (n = 23)	Stent related Functional disorders	Obstruction/ileus Infectious complications Surgery related Cardiovascular events	5
Faragher, I.G. (Australia, 2008) [20]	Retrospective, left-sided obstructive unresectable CRC. SEMS group: M/F = 17/12, median age 70 years (range, 44–95). Surgery group: M/F = 16/10, median age 67 years (range, 33–90).	Bowel obstruction due to incurable left-sided CRC can be effectively palliated using SEMSs with a low complication rate.	N = 55 SEMS (n = 29) Surgery (n = 26)	Stent related Obstruction/ileus Functional disorders Cardiovascular events	Infectious complications Cardiovascular events Organ failure Thromboembolic events Stoma related	6
Finlayson, A. (New Zealand, 2016) [21]	Retrospective, patients with obstructing or near-obstructing stage IV CRC. SEMS group: M/F = 42/23, median age 76 years. Surgery group: M/F = 34/29, median age 71 years.	SEMSs have high clinical success rates and low complications rate. No difference in mortality between groups.	N = 128 SEMS (n = 65) Surgery (n = 63)	Stent related Obstruction/ileus Bleeding/anemia	-	5
Fiori, E. (Italy, 2012) [22]	RCT, patients with stage IV unresectable rectosigmoid cancer and symptoms of chronic subacute obstruction.	No significant difference in survival between groups.	N = 22 SEMS (n = 11) Surgery SF/IB (n = 11)	Obstruction/ileus Bleeding/anemia	Functional disorders Stoma related	Moderate risk
van Hooft, J.E. (Netherlands, 2008) [23]	RCT, stage IV left-sided CRC.	High rate of perforation in the SEMS group.	N = 21 SEMS (n = 11) Surgery (n = 10)	Stent related Obstruction/ileus Functional disorders	Obstruction/ileus	Low risk
Johnson, R. (United Kingdom, 2004) [24]	Retrospective, patients with obstructive CRC who underwent SEMS placement. Historical control of patients who underwent surgical stoma formation. Surgical patients were younger (*p* = 0.0065) and had less co-morbidity (*p* = 0.04).	Both groups experienced relief of obstructive symptoms. There were no differences in survival or mortality. Selected patients benefit from SEMS with relief of obstructive symptoms and no adverse effect on survival.	N = 36 SEMS (n = 18) Surgery SF/IB (n = 18)	Stent related Obstruction/ileus	Infectious complications	6
Law, W.L. (Hong Kong, 2003) [25]	Prospective, patients with incurable obstructing left-sided CRC. SEMS group: M/F = 20/10, median age 75 years (range, 36–98). Surgery group: M/F = 20/11, median age 70 years (range, 38–89). Similar medical comorbidities between groups.	SEMSs should be considered in patients with incurable obstructing CRC due to shorter hospital stay and lower incidence of stoma formation.	N = 61 SEMS (n = 30) Surgery (n = 31)	Stent related Functional disorders Cardiovascular events	Infectious complications Cardiovascular events Organ failure Thromboembolic events	8
Lee, H.J. (South Korea, 2011) [26]	Retrospective, patients with incurable obstructive CRC, with or without previous chemotherapy, ASA I-III. SEMS group: M/F = 47/24, median age 64 years (range, 26–87). Surgery group: M/F = 47/26, median age 62 years (range, 29–88).	Long-term outcomes and complications were comparable between groups. Stent-related late complications were manageable with endoscopic treatment. SEMS should also be recommended to patients with a longer life expectancy.	N = 144 SEMS (n = 71) Surgery (n = 73)	Stent related Obstruction/ileus	Obstruction/ileus Bleeding/anemia Infectious complications Organ failure Thromboembolic events	7
Lee, W.S. (South Korea, 2012) [27]	Prospective, patients with stage IV obstructive CRC and prior chemotherapy. SEMS was chosen for patients with higher ASA scores (III–IV), liver insufficiency due to diffuse metastatic disease and patient’s refusal of surgery.	SEMS shortened the hospital stays, avoided need for colostomy, and allowed chemotherapy to be administered earlier.	N = 88 SEMS (n = 36) Surgery RPT (n = 52)	Stent related Obstruction/ileus	Obstruction/ileus Bleeding/anemia Surgery related Infectious complications	7
Mahfouz, M.F. (Egypt, 2022) [28]	Retrospective, emergency intervention for acute malignant left-sided colonic obstruction and metastatic disease. SEMS group: M/F = 26/9, median age 69 years (range, 63–79). Surgery group: M/F = 21/8, median age 67 years (range, 61–75).	SEMS provided comparable efficacy and safety to surgery and faster recovery.	N = 64 SEMS (n = 35) Surgery RPT (n = 29)	Obstruction/ileus Bleeding/anemia Infectious complications	Obstruction/ileus Bleeding/anemia Infectious complications	7
Pattarajierapan, S. (Thailand, 2022) [29]	Retrospective, patients with incurable stage IV CRC.	SEMS was safe and effective.	N = 202 SEMS (n = 105) Surgery SF/IB (n = 97)	Stent related Obstruction/ileus	Functional disorders Bleeding/anemia Infectious complications Surgery related Stoma related	7
Ptok, H. (Germany, 2006) [30]	Prospective, non-randomized, patients with unresectable CRC. Study groups differed significatively in terms of age (*p* = 0.020) and ASA score (*p* = 0.012).	In comparison with SEMS, surgical palliative intervention for incurable CRC, especially in older patients, provided no significant advantages.	N = 78 SEMS (n = 40) Surgery (n = 38)	Stent related Obstruction/ileus	-	8
Seoane, A. (Spain, 2019) [31]	Prospective, patients with stage IV obstructive CRC. SEMS group: M/F = 29/17, median age 68.9 years. Surgery group: M/F = 28/21, median age 65.2 years. Surgical group had a higher proportion of prior chemotherapy (89.4% vs. 69.8%, *p* = 0.02). No differences between groups in ASA scores.	The key to treatment resided in assessing cases on an individual basis. SEMS may be considered for patients with a higher ASA score who are not eligible for chemotherapy due to a shorter life expectancy.	N = 95 SEMS (n = 46) Surgery RPT (n = 49)	Stent related Obstruction/ileus Functional disorders	Obstruction/ileus Bleeding/anemia Infectious complications	6
Suarez, J. (Spain, 2010) [32]	Retrospective, patients with stage IV obstructive CRC. Both groups were comparable regarding age, ASA score, prior chemotherapy, tumor location and presence of metastatic disease.	SEMS should be considered when tumors are non-resectable or when the patient is not a candidate for systemic therapy due to co-morbidities.	N = 98 SEMS (n = 45) Surgery (n = 53)	Stent related Obstruction/ileus Functional disorders	Bleeding/anemia Infectious complications Surgery related Stoma related Cardiovascular events Organ failure	7
Vemulapalli, R. (United States, 2009) [33]	Retrospective, patients with incurable obstructive CRC. No differences between groups in age.	SEMS should be first-line therapy in patients with acute malignant obstruction due to lower complication and mortality rates and shorter hospital stay. Surgery was an option for patients in whom SEMS was unsuccessful or contraindicated.	N = 123 SEMS (n = 53) Surgery (n = 70)	Stent related Obstruction/ileus	Obstruction/ileus Infectious complications Surgery related Cardiovascular events Organ failure Thromboembolic events	5
Zhang, Y.X. (China, 2012) [34]	Retrospective, patients with incurable obstructive CRC. No differences between groups in age.	SEMS was suitable for patients with poor general condition. No significant difference in the main complications between SEMS and surgery. The long-term efficacy of SEMS was worse than that of surgery.	N = 186 SEMS (n = 97) Surgery (n = 89)	Stent related Obstruction/ileus Infectious complications Thromboembolic events	Bleeding/anemia Infectious complications Surgery related Organ failure Thromboembolic events	6

RCT—randomized controlled trial; N—total number of patients in each study included; n—number of patients in each arm of included studies; SEMS—self-expanding metal stent; SF/IB—stoma formation/internal bypass; RPT—resection of primary tumor; NOS—Newcastle–Ottawa Scale; ASA—American Society of Anesthesiologists class.

**Table 3:** Subgroup analysis for high heterogeneity outcomes.

Outcome	Variable for Subgroup Analysis	Nr. of Studies	Nr. of Patients SEMS/Surgery	*I* ^2^	OR/SMD [95%CI]	*p*-Value
**Early Complications**						
Year of publication	≤2010	4	138/159	68%	0.25 [0.07, 0.94]	0.04
	**>2010**	**7**	**438/389**	**39%**	**0.42 [0.25, 0.71]**	**0.001**
Type of study	Prospective	2	151/146	88%	0.20 [0.02, 2.57]	0.22
	**Retrospective**	**7**	**403/381**	**41%**	**0.33 [0.19, 0.55]**	**<0.0001**
	RCT	2	22/21	46%	1.59 [0.10, 24.41]	0.74
Location of CRC	Left-sided CRC	4	86/76	70%	0.47 [0.08, 2.76]	0.40
	NOS CRC	7	490/472	54%	0.32 [0.18, 0.56]	<0.0001
Type of surgical intervention	SF/IB	2	116/108	0%	0.63 [0.26, 1.51]	0.3
	**RPT**	**2**	**106/102**	**0%**	**0.44 [0.23, 0.87]**	**0.02**
	NOS Surgery	7	354/338	69%	0.26 [0.11, 0.61]	0.002
**Late Complications**						
Year of publication	**≤** **2010**	**4**	**138/159**	**14%**	**3.03 [1.39, 6.56]**	**0.005**
	>2010	6	403/360	80%	1.92 [0.76, 4.86]	0.17
Type of study	Prospective	2	151/146	93%	1.46 [0.08, 26.78]	0.8
	**Retrospective**	**6**	**368/352**	**0%**	**2.73 [1.79, 4.15]**	**<0.00001**
	RCT	2	22/21	63%	3.21 [0.19, 54.97]	0.42
Location of CRC	Left-sided CRC	3	51/47	28%	1.83 [0.55, 6.09]	0.32
	NOS CRC	7	490/472	79%	2.47 [1.08, 5.65]	0.03
Type of surgical intervention	**SF/IB**	**2**	**116/108**	**14%**	**0.44 [0.19, 1.00]**	**0.05**
	RPT	1	71/73	NA	2.36 [1.09, 5.12]	0.03
	**NOS Surgery**	**7**	**354/338**	**0%**	**3.34 [2.11, 5.29]**	**<0.00001**
**Length of Hospital Stay**						
Year of publication	≤2010	1	40/38	NA	−5.15 [−6.10, −4.21]	<0.00001
	>2010	6	323/217	88%	−1.23 [−1.85, −0.62]	<0.00001
Type of study	Prospective	2	76/49	99%	−2.63 [−7.55, 2.28]	0.29
	Retrospective	4	276/195	82%	−1.08 [−1.56, −0.60]	<0.00001
	RCT	1	11/11	NA	−5.74 [−7.79, −3.69]	<0.00001
Location of CRC	Left-sided CRC	2	46/40	92%	−3.71 [−7.41, −0.01]	0.05
	NOS CRC	5	317/215	95%	−1.48 [−2.45, −0.51]	0.003
Type of surgical intervention	SF/IB	1	11/11	NA	−5.74 [−7.79, −3.69]	<0.00001
	RPT	2	71/40	93%	−1.05 [−2.82, 0.73]	0.25
	NOS Surgery	4	281/255	96%	−1.82 [−2.94, −0.69]	0.002

OR—odds ratio (calculated for dichotomous variables using Mantel–Haenszel method and random-effects models); SMD—standard mean difference (calculated for continuous variables using inverse variance method and random-effects models); NA—not applicable; CRC—colorectal cancer; RCT—randomized controlled trial; NOS—not otherwise specified; SF/IB—stoma formation/internal bypass; RPT—resection of primary tumor; SEMS—self-expanding metal stent; 95%CI—95% confidence interval.

## Data Availability

All studies included in this review are available online.

## Abbreviations

The following abbreviations are used in this manuscript:

SEMS	Self-expanding metal stent
CRC	Colorectal cancer
AO-CRC	Advanced obstructive colorectal cancer
SF/IB	Stoma formation or internal bypass
RPT	Resection of primary tumor
OR	Odds ratio
SMD	Standard mean difference
95%CI	95% confidence interval
RCT	Randomized controlled trial
NA	Not applicable
NOS	Not otherwise specified
